# Higher intakes of fiber, total vegetables, and fruits may attenuate the risk of all-cause and cause-specific mortality: findings from a large prospective cohort study

**DOI:** 10.1186/s12937-023-00883-4

**Published:** 2023-11-17

**Authors:** Zeinab Ghorbani, Morvarid Noormohammadi, Asma Kazemi, Hossein Poustchi, Akram Pourshams, Fahimeh Martami, Maryam Hashemian, Reza Malekzadeh, Azita Hekmatdoost

**Affiliations:** 1 Cardiovascular Diseases Research Center, Department of Cardiology, Heshmat Hospital, School of Medicine, Guilan University of Medical Sciences, Rasht, Iran; 2 Digestive Oncology Research Center, Digestive Diseases Research Institute, Tehran University of Medical Sciences, Tehran, Iran; 3 Department of Clinical Nutrition, School of Medicine, Guilan University of Medical Sciences, Rasht, Iran; 4 Department of Nutrition, School of Public Health, Iran University of Medical Sciences, Tehran, Iran; 5 Nutrition Research Center, School of Nutrition and Food Sciences, Shiraz University of Medical Sciences, Shiraz, Iran; 6 Liver and Pancreatobiliary Diseases Research Center, Digestive Diseases Research Institute, Tehran University of Medical Sciences, Tehran, Iran; 7 School of Nutritional Sciences and Dietetics, Tehran University of Medical Sciences, Tehran University of Medical Sciences, Tehran, Iran; 8 School of Arts & Sciences, Utica University, Utica, NY USA; 9 Department of Clinical Nutrition and Dietetics, Faculty of Nutrition and Food Technology, National Nutrition and Food Technology Research Institute, Shahid Beheshti University of Medical Sciences, Tehran, Iran

**Keywords:** Death risk, Dietary fiber, Cardiovascular disorders, Cancer, Golestan cohort study

## Abstract

**Background:**

Although studies have reported an inverse association between fruits, vegetables, and fiber consumption and all-cause and cause-specific mortality, the issue remains incompletely defined in the Middle Eastern population.

**Aims:**

The current study aimed to investigate the association between dietary fiber, fruit, and vegetable intake and all-cause and cause-specific mortality.

**Methods:**

A total of 48632 participants (mean age = 52years), 57.5% (*n* = 27974) women and 42.5% (*n* = 20658) men, were recruited from an ongoing large-scale prospective cohort study (the Golestan Cohort Study (GCS)), in the north of Iran. Using a validated semi-quantitative 116-item food questionnaire, dietary intakes were collected. Hazard ratios (HRs) and 95% confidence intervals (95%CIs) of all-cause and cause-specific mortality were reported.

**Results:**

After approximately 14 years of follow-up, 10,774 deaths were recorded. In the fully adjusted model, compared to those in the lowest quintile of intake, those in the second and third quintiles of dietary fiber intake had a 7%-10% reduction in risk of all-cause mortality, and a 15%-17% reduction in the risk of mortality from other causes. Increasing consumption of fruits was also associated with a decreased risk of mortality for all-cause mortality by 9%-11%, and all cancer by 15–20%. Further, those in the third and fourth quintiles of vegetables intake had 11%-12% lower risk for CVD mortality.

**Discussion:**

The results from the GCS further support the current recommendations on following a healthy diet containing proper amounts of fiber, vegetables, and fruits, as health-protective dietary items.

**Conclusions:**

Higher intake of dietary fiber, fruits, and vegetables has the potential to reduce both overall and cause-specific mortality rates. However, additional cohort studies with larger sample size and long-term follow-up durations are required to establish these findings.

**Supplementary Information:**

The online version contains supplementary material available at 10.1186/s12937-023-00883-4.

## Introduction

Globally, due to being among the ten leading causes of death, years of life lost, and DALYs, cardiovascular disease (CVD) and cancer are listed as major public health concerns [1]. Based on the Systematic Analysis for the Global Burden of Disease (GBD) Study 2019, 23.6 million new cancer cases and 10.0 million cancer deaths were estimated globally in 2019 [2]. The World Health Organization (WHO) has reported that in 2019, approximately 17.9 million people worldwide died from CVD, which accounted for 32% of all global deaths [3]. Some important risk factors for CVD and cancer include cigarette smoking, obesity, overweight, and an unbalanced diet [4]. Dietary patterns are associated with chronic diseases, including CVD and cancer, by preventing or delaying disease occurrence [5]. The Mediterranean dietary pattern, high in fruits and vegetables, is proposed to be the most cardioprotective dietary pattern and a powerful approach for fighting cancer related mortality [6, 7].

Several research studies have investigated the association between consumption of fruits and vegetables with cause-specific mortality focused on CVD [8–10], stroke [11] and cancer [12, 13]. Overall, based on the evidence of some extensive cohort studies, adequate intake of fruits and vegetables was inversely related to all-cause mortality [13–17], including the European Prospective Investigation into Cancer and Nutrition (EPIC) study [18].

Similarly, these results are consistent with regards to fiber intake as shown by the EPIC study [19], the Japan Public Health Center-based study [20], the large cohort study among US adults [21], the Prevención con Dieta Mediterránea (PREDIMED) study [22], and the "Seguimiento Universidad de Navarra" (SUN) project [23].

There are a number of ways in which eating fruits and vegetables can lower the risk of CVD, cancer and death. One can be through the dietary fiber found in these foods, which would be helpful in reducing blood pressure, improve cholesterol levels, and increase insulin sensitivity [24]. Increasing the total consumption of fruits and vegetables, rich in bioactive compounds such as polyphenols, fiber, vitamins, and minerals, has been suggested to reduce CVD and cancer risk by exerting antioxidant, anti-inflammatory, and antithrombotic effects [6, 7]. The optimal fruits and vegetables consumption level to reduce the risk of chronic diseases and mortality is still unclear. However, WHO recommended for a minimum daily intake of 400 g of fruits and vegetables to prevent diet-related chronic diseases [25].

However, there has been some controversy regarding the association between fruits and vegetables and mortality as some studies showed no clear association [26–28]. Although results from the previous cohort studies tend to suggest that inverse associations between fruit and vegetables and mortality, based on the reports of two systematic reviews, most of these research were conducted in U.S and Europe countries [14, 17]. To date, a few well-powered studies assessed this association in Asian populations [9, 29, 30] and so far, there were no prospective cohort data from west Asia countries, including Iran [14]. Furthermore, there were several different aspects between Western and Asian countries, including dietary patterns [31], the prevalence of obesity [32], and the distribution of disease in the mortality rate [32, 33]. Because of these reasons, the firm establishment of the association mentioned above in the Asian population, including Iran, needs further investigation. On the other hand, a limitation of much of the previous data is that they have been conducted in special populations (like physicians and nurses) who might be health-conscious, which may have biased the results [34, 35].

As a large prospective population-based cohort study in Iran, the Golestan Cohort Study (GCS), that predominantly aimed to investigate the risk factors involved in the etiology of esophageal cancer (EC), enrolled a total of 50,045 participants in a high risk region (Golestan province, located in the north of Iran) in 2004. The subjects are being followed-up until now [36]. Thus, the current investigation is designed to explore the association between dietary intake of fiber, fruits and vegetables and all-causes and cause-specific mortality among Iranian general population who participated in the GCS.

## Methods & materials

### Study design and population

The Golestan Cohort Study (GCS) is an ongoing prospective cohort study that was initially designed to investigate the cause of esophageal cancer (EC) in Golestan, where the prevalence of EC is extremely high, as reported previously. Further details about the study population and data collection process have been described and published before [36]. In summary, GCS included 50,045 adult participants aged between 40 and 87 years who were recruited between 2004 and 2008. Participants enrolled from Gonbad City and 326 rural villages (20% urban areas and 80% rural cohort) in Golestan province, northeastern Iran. Subjects with missing data on the food frequency questionnaire (FFQ), demographics and anthropometric measurements, education, socio-economic status (SES), smoking, alcohol and opium use, and who reported a history of diabetes and/or prevalent cancers at the baseline were excluded. Participants with extremely low or high energy intakes (< 500 or > 5000 kcal/ day) and those with an unreasonable BMI (less than 15 or more than 50 kg/m2) were also excluded. The final sample that is used in the present analysis comprises 48,633 participants (20,658 men and 27,975 women).

The study was conducted according to the guidelines laid down in the Declaration of Helsinki and all procedures involving human subjects/patients were approved by the Institutional Review Boards of the Digestive Disease Research Center of Tehran University of Medical Sciences, the US National Cancer Institute (NCI), and the World Health Organization International Agency for Research on Cancer (IARC) [ethic number: 81/15]. All participants gave written informed consent before participation in the study.

### Dietary assessment

Dietary data collection in GCS has been applied using A validated semi-quantitative 116-item FFQ, which was used to assess the participants’ eating habits during the last years [37]. Trained, skilled nutritionists completed FFQ throughout a face-to-face interview. Participants were asked to report the frequency and portion size of consumption of each food item on a daily, weekly, monthly, yearly, or never basis. Daily consumption of each food item was calculated by multiplying the frequency of consumption by average portion sizes. Then, the reported portion size of each consumed food was converted to grams. Fruits questions referred to watermelon, melon, cucumbers, apples, apricot, yellow plum, cherries, sour cherry, nectarine, peach, pears, fig, oranges, tangerine, lemons, date palm, and grapes. Potato, tomato (raw & cooked), tomato paste, eggplant, onion, mixed vegetables, green beans, peas, broad bean, zucchini, lettuce, cooked squash, radish, cabbage, mushroom, green pepper, bell pepper, carrot, garlic, and pickled garlic were captured as vegetables on this FFQ. Energy and nutrient content were analyzed using Nutritionist software version IV (Nutritionist IV, Version 3.5.2).

### Assessment of potential confounders

Trained physicians interviewed all participants to complete a structured lifestyle questionnaire comprising socio-demographic Characteristics, history of alcohol drinking, tobacco, opium, and cigarette smoking. Furthermore, all participants recorded their wealth score (a surrogate of SES 18, calculated from appliance ownership). Details about the medical history of diagnosed diseases and medications were also fulfilled. Anthropometric variable measurements such as weight, height, body mass index, and waist and hip circumferences were measured by trained research staff.

### Mortality follow-up

Details about the procedures of recording and confirmation of the causes of death in this cohort study have been reported elsewhere [36]. In brief, since access to 98% of GCS participants was possible through telephone calls, telephone contacts were considered as the first long-term follow-up method. During each phone call or home visit (in case it is not possible to contact the person or their relatives after seven attempts), the GCS team fills out a case review questionnaire and documents the participant's current condition and any instances of illness or hospital admissions since the last contact. The participants are also asked if they have any intention to migrate in the near future. If there is a report of death, cancer, or upper gastrointestinal (UGI) endoscopy, personal visits are made by the GCS team to the subject’s home and the medical centers where any significant diagnostic or therapeutic procedures were conducted. They gather all relevant clinical or pathology reports, hospital admission documents, and any available tumor samples. In the case of deceased subjects, a verbal autopsy is also conducted [36].

Regarding ascertainment of the causes of death, to ensure accuracy, two external internists separately evaluate all available clinical documents and assign a disease code (according to the international classification of diseases and related health problems (ICD_10_)) and date to each outcome that has been reported for the cohort participants. After comparing these recorded codes, if any discrepancies are noted, a superior internist re-checks the documents and provides the final decision on the code. Specifically, as UGI cancer outcomes hold particular significance in the study, they undergo additional scrutiny by an International Endpoint Review Committee (IERC) composed of experts from the Digestive Disease Research Center (DDRC), International Agency for Research on Cancer (IARC) and US National Cancer Institute (NCI) [36].

In the present analysis, causes of death were categorized as all-cause death and deaths due to all-cancers, GI-cancers, CVDs, and other causes.

### Statistical analysis

The studied subject’s characteristics and dietary intakes at baseline were compared across quintiles of dietary fiber using linear regression analysis. Then after, the corresponding data were presented as means, standard deviations (SDs) (or median (interquartile range, IQR)), and frequencies (n and percentages) in the case of continuous and categorical variables, respectively.

The studied population was divided into quintiles according to the daily intakes of total fiber, vegetables, and fruits. A new continuous variable was generated to explore the linear trends for all-cause and cause-specific mortality risk across the quintiles based on calculating the median value for each quintile, replacing it with each quintile, and then adding this as a continuous variable in the model to test for trend.

In order to explore the relationship between dietary fiber, fruits, and vegetables consumption and all-cause and cause-specific death risk, Cox regression models were conducted.

Participants’ age at the end of follow-up (July 2022), death, or loss to follow-up or, either recorded earliest, deducted from their age at participation to study (time zero) was used as the time scale for the Cox regression analysis. The initial Cox regression models (Model 1) were controlled for age at cohort baseline, gender, and total daily energy intake (kcal/day). In the fully adjusted Cox regression models. The participants’ residential area, level of physical activity, educational level, BMI, smoking status (ever-smoker, never-smoker, pack-years of cigarettes), wealth score (WS), using opium and alcohol, having a past medical history of diabetes, and dietary intakes (g/day), including red and processed meat, fish, poultry, dairy products, refined grains, and total fats. Accordingly, all-cause and cause-specific hazard ratios (HRs) and the corresponding 95% confidence intervals (95% CIs) were described.

Due to the probable confounding effects of chronic disorders and past medical histories on dietary consumption and mortality risk, we decided to exclude those subjects suffering from chronic diseases, extreme BMI, the participants with a history of smoking, opium or alcohol use at study enrollment in sensitivity analysis. The deaths recorded throughout the initial two years of the present cohort follow-up were excluded in an additional sensitivity analysis. All statistical analyses were performed with STATA software (version 14, STATA Corp, College Station, TX, USA). All *P-*values were two-sided, and *P* < 0.05 were considered the statistical significance level.

## Results

### Baseline characteristics

After excluding participants with incomplete baseline data, a total of 48632 subjects, with a mean age of 52 years, were enrolled in the current investigation; Of whom, about 57.5% (*n* = 27974) were women and 42.5% (*n* = 20658) were men. After 13.8 years of follow-up on average, 10,774 deaths were recorded, of which 3,868 deaths were due to CVDs, 1,826 due to cancer (with 985 GI cancer death), and 3,160 death cases were due to other causes (e.g., respiratory and infectious diseases).

Table 1 describes the baseline characteristics and dietary intakes of studied participants according to the first and last quintiles of dietary fiber, fruits and vegetable. Compared to the subjects in the first quintile, individuals in the highest quintile of dietary fiber, fruits and vegetables were more likely to be men, younger, more rural, less hypertensive, or to have higher educational levels, wealth score (socio-economic status), and BMI; At the same time, they were more likely to smoke cigarettes, and be alcohol drinkers. Regarding the physical activity level and dietary intakes, the participants with higher fiber consumption tended to be more active, have higher daily intakes of energy and carbohydrate and lower daily intakes of total fat and total protein. The participants with higher fruits consumption tended to be less active, have higher daily intakes of energy, protein and total fat and lower intake of carbohydrate. Furthermore, those with higher vegetables consumption were more likely to be active and have greater daily intakes of energy and total fat and lower protein and carbohydrate consumption (Table 1).

**Table 1:** Baseline characteristics of studied subjects according to the first and last quintiles of dietary fiber, fruits and vegetables intakes in a large prospective cohort study with 14 years of follow-up

		**Fiber**		***P*-value**	**Fruit**		***P*-value**	**Vegetable**		***P*-value**
		**Quintile 1**	**Quintile 5**		**Quintile 1**	**Quintile 5**		**Quintile 1**	**Quintile 5**	
		*N* = 9727	*N* = 9726		*N* = 9727	*N* = 9726		*N* = 9727	*N* = 9726	
**Age** (Mean ± SD)		53.84 ± 9.48	51.21 ± 8.51	< 0.001	53.40 ± 9.28	51.45 ± 8.67	< 0.001	53.33 ± 9.48	51.97 ± 8.65	< 0.001
**Gender** (n (%)	Female	7205 (74.07%)	3822 (39.3%)	< 0.001	6464 (66.45%)	4418 (45.42%)	< 0.001	5831 (59.95%)	5382 (55.33%)	< 0.001
	Male	2522 (25.93%)	5904 (60.7%)		3263 (33.55%)	5308 (54.58%)		3896 (40.05%)	4345 (44.67%)	
**Education** (n (%)	Illiterate	7898 (81.2%)	5849 (60.14%)	< 0.001	8349 (85.83%)	5325 (54.75%)	< 0.001	7699 (79.15%)	5708 (58.68%)	< 0.001
	Diploma or Less	1745 (17.94%)	3513 (36.12%)		1357 (13.95%)	3872 (39.81%)		1964 (20.19%)	3588 (36.89%)	
	University	84 (0.86%)	364 (3.74%)		21 (0.22%)	529 (5.44%)		64 (0.66%)	431 (4.43%)	
**Ethnicity** (n (%)	Turkmen	7040 (72.38%)	7108 (73.08%)	0.27	5969 (61.37%)	7477 (76.88%)	< 0.001	7302 (75.07%)	6350 (65.28%)	< 0.001
	non-Turkmen	2687 (27.62%)	2618 (26.92%)		3758 (38.63%)	2249 (23.12%)		2425 (24.93%)	3377 (34.72%)	
**Body mass index category** (n (%)	Normal	4558 (46.96%)	3533 (36.37%)	< 0.001	4976 (51.24%)	3107 (32.01%)	< 0.001	4556 (46.92%)	3188 (32.83%)	< 0.001
	Overweight	2944 (30.33%)	3548 (36.52%)		2861 (29.46%)	3661 (37.72%)		3145 (32.39%)	3543 (36.48%)	
	Obese	2205 (22.72%)	2633 (27.11%)		1875 (19.31%)	2938 (30.27%)		2009 (20.69%)	2981 (30.69%)	
**Cigarette smoking** (n (%)	Never	8527 (87.66%)	7443 (76.53%)	< 0.001	8338 (85.72%)	7637 (78.52%)	< 0.001	8128 (83.56%)	7919 (81.41%)	< 0.001
	Ever	1200 (12.34%)	2283 (23.47%)		1389 (14.28%)	2089 (21.48%)		1599 (16.44%)	1808 (18.59%)	
**Opiate** (n (%)	Never	7954 (81.77%)	7926 (81.49%)	0.61	7965 (81.89%)	8129 (83.58%)	0.002	7844 (80.64%)	8276 (85.08%)	< 0.001
	Ever	1773 (18.23%)	1800 (18.51%)		1762 (18.11%)	1597 (16.42%)		1883 (19.36%)	1451 (14.92%)	
**Alcohol** (n (%)	Never	9518 (97.85%)	9154 (94.12%)	< 0.001	9574 (98.43%)	9053 (93.08%)	< 0.001	9537 (98.05%)	9154 (94.11%)	< 0.001
	Ever	209 (2.15%)	572 (5.88%)		153 (1.57%)	673 (6.92%)		190 (1.95%)	573 (5.89%)	
**History of Diabetes** (n (%)	No	8933 (91.84%)	9014 (92.68%)	0.03	9190 (94.48%)	8971 (92.24%)	< 0.001	8975 (92.27%)	8914 (91.64%)	0.11
	Yes	794 (8.16%)	712 (7.32%)		537 (5.52%)	755 (7.76%)		752 (7.73%)	813 (8.36%)	
**History of Hypertension** (n (%)	No	7282 (74.86%)	8181 (84.11%)	< 0.001	7784 (80.02%)	7921 (81.44%)	0.01	7491 (77.01%)	7837 (80.57%)	< 0.001
	Yes	2445 (25.14%)	1545 (15.89%)		1943 (19.98%)	1805 (18.56%)		2236 (22.99%)	1890 (19.43%)	
**Wealth Score quartile** (n (%)	Q1	3069 (31.55%)	2592 (26.65%)	< 0.001	4266 (43.86%)	1764 (18.14%)	< 0.001	3289 (33.81%)	2081 (21.39%)	< 0.001
	Q2	2435 (25.03%)	2065 (21.23%)		3059 (31.45%)	1556 (16%)		2568 (26.4%)	1840 (18.92%)	
	Q3	2245 (23.08%)	2272 (23.36%)		1754 (18.03%)	2166 (22.27%)		2335 (24.01%)	2211 (22.73%)	
	Q4	1978 (20.34%)	2797 (28.76%)		648 (6.66%)	4240 (43.59%)		1535 (15.78%)	3595 (36.96%)	
**Residential Area** (n (%)	Rural	8075 (83.02%)	7011 (72.09%)	< 0.001	8538 (87.78%)	6193 (63.67%)	< 0.001	8591 (88.32%)	6122 (62.94%)	< 0.001
	Urban	1652 (16.98%)	2715 (27.91%)		1189 (12.22%)	3533 (36.33%)		1136 (11.68%)	3605 (37.06%)	
**Physical Activity** (n (%)	Low	3498 (35.96%)	4287 (44.08%)	< 0.001	3306 (33.99%)	4459 (45.85%)	< 0.001	4070 (41.84%)	3569 (36.69%)	< 0.001
	Medium	3965 (40.76%)	2612 (26.86%)		3746 (38.51%)	2866 (29.47%)		3337 (34.31%)	3305 (33.98%)	
	High	2264 (23.28%)	2827 (29.07%)		2675 (27.5%)	2400 (24.68%)		2320 (23.85%)	2853 (29.33%)	
**Dietary intakes** Median (IQR)										
**Energy** (kcal/d)		1483.14 (1247.45–1702.91)	2806.15 (2549.62–3151.68)	< 0.001	1807.99 (1461.49–2141.54)	2490.97 (2132.49–2881.73)	< 0.001	1760.61 (1431.5–2083.82)	2500.21 (2148.65–2881.38)	< 0.001
**Carbohydrate** (g/d)		52 (46.69–57.12)	59.81 (56.22–63.37)	< 0.001	57.91 (52.78–62.75)	57.71 (53.42–61.61)	0.01	58.2 (52.95–62.91)	56.15 (51.92–60.18)	< 0.001
**Total fat** (g/d)		35.62 (31.04–39.95)	29.19 (26.23–32.17)	< 0.001	30.59 (26.45–34.88)	30.88 (27.61–34.5)	< 0.001	29.78 (25.8–34.05)	32.37 (28.92–35.92)	< 0.001
**Total Protein** (g/d)		14.01 (12.14–16.36)	13.51 (12.37–14.87)	< 0.001	13.54 (12.06–15.34)	13.81 (12.5–15.41)	< 0.001	14.11 (12.66–16.01)	13.69 (12.28–15.36)	< 0.001

### Dietary fiber intake and all-cause and cause-specific mortality

Tables 2, 3 and 4 presents the Cox multiple regression analysis findings on the association between dietary intakes of fiber, total fruits and vegetables, and hazard ratios (HRs) of all-cause and cause-specific mortality.

**Table 2:** Hazard ratios for all-cause and cause-specific mortality, according to quintiles of dietary fiber intake in a large prospective cohort study with 14 years of follow-up

	**Quintile of Fiber**					**P for linear trend**
	**1**	**2**	**3**	**4**	**5**	
**Median intake**	13.66	18.94	22.49	25.67	31.37	
**Person-years of follow-up**	129728.50	133316.21	134656.65	136001.09	137861.73	
**All-cause mortality**						
**Mortality rate (per 1000 person-years)**	19.9	16.4	14.6	14.5	15	
**Model 1, HR (95% CI)**	1	0.87(0.82, 0.92)	0.82 (0.77, 0.87)	0.83 (0.78, 0.88)	0.79 (0.74, 0.84)	< 0.001
**Model 2, HR (95% CI)**	1	0.91(0.86, 0.96)	0.88(0.83, 0.93)	0.91(0.86, 0.97)	0.87(0.82, 0.92)	< 0.001
**Model 3, HR (95% CI)**	1	0.93(0.87, 0.99)	0.90(0.83, 0.97)	0.95(0.87, 1.03)	0.92(0.82, 1.03)	0.30
**All cancer cause**						
**Mortality rate (per 1000 person-years)**	3.1	2.8	2.4	2.7	2.8	
**Model 1, HR (95% CI)**	1	0.93(0.80, 1.07)	0.82(0.71, 0.96)	0.95(0.82, 1.10)	0.92(0.80, 1.06)	0.40
**Model 2, HR (95% CI)**	1	0.95(0.82, 1.10)	0.86(0.74, 0.998)	1.01(0.88, 1.17)	0.99(0.85, 1.15)	0.79
**Model 3, HR (95% CI)**	1	0.91(0.78, 1.07)	0.81(0.67, 0.98)	0.94(0.76, 1.16)	0.87(0.66, 1.15)	0.45
**GI cancer cause**						
**Mortality rate (per 1000 person-years)**	1.7	1.4	1.2	1.4	1.6	
**Model 1, HR (95% CI)**	1	0.82(0.67, 0.99)	0.71(0.58, 0.87)	0.87(0.72, 1.06)	0.90(0.74, 1.09)	0.52
**Model 2, HR (95% CI)**	1	0.84(0.69, 1.01)	0.74(0.60, 0.91)	0.93(0.76, 1.13)	0.98(0.80, 1.19)	0.81
**Model 3, HR (95% CI)**	1	0.80(0.64, 1.00)	0.69(0.53, 0.89)	0.85(0.64, 1.12)	0.85(0.59, 1.22)	0.53
**CVD cause**						
**Mortality rate (per 1000 person-years)**	7.1	5.8	5.3	5.2	5.4	
**Model 1, HR (95% CI)**	1	0.89(0.80, 0.97)	0.86(0.78, 0.95)	0.89(0.81, 0.99)	0.88(0.80, 0.98)	0.043
**Model 2, HR (95% CI)**	1	0.93(0.84, 1.02)	0.93(0.84, 1.03)	0.98(0.89, 1.09)	0.96(0.87, 1.06)	0.81
**Model 3, HR (95% CI)**	1	0.97(0.87, 1.08)	0.99(0.86, 1.12)	1.07(0.92, 1.24)	1.09(0.90, 1.32)	0.22
**Other cause**						
**Mortality rate (per 1000 person-years)**	6.1	4.6	4.4	4.2	4.4	
**Model 1, HR (95% CI)**	1	0.79(0.71, 0.87)	0.79(0.71, 0.88)	0.79(0.70, 0.88)	0.77(0.69, 0.86)	< 0.001
**Model 2, HR (95% CI)**	1	0.84(0.75, 0.93)	0.87(0.78, 0.97)	0.88(0.79, 0.99)	0.86(0.77, 0.96)	0.06
**Model 3, HR (95% CI)**	1	0.83(0.74, 0.94)	0.85(0.74, 0.98)	0.87(0.74, 1.02)	0.84(0.68, 1.03)	0.21

Model 1: Gender and age adjusted

Model 2: Additionally adjusted for Educational level, Ethnicity, Body mass index, Cigarette smoking, Opiate use, Alcohol consumption, Having a history of diabetes, Having a history of hypertension, Wealth score, Residential area, and Physical activity level

Model 3: Additionally adjusted for Daily energy intake (kcal/d), and Energy-adjusted carbohydrate, protein and fat consumption (g/d)

**Table 3:** Hazard ratios for all-cause and cause-specific mortality, according to quintiles of fruits intake in a large prospective cohort study with 14 years of follow-up

	**Quintile of Fruit**					**P for linear trend**	
	**1**	**2**	**3**	**4**	**5**		
**Median intake**	41.26	81.74	120.86	175.81	300.05		
**Person-years of follow-up**	129310.88	131690.17	134424.09	135841.02	140298.02		
**All-cause mortality**							
**Mortality rate (per 1000 person-years)**	19.6	17	14.4	14.9	14.6		
**Model 1, HR (95% CI)**	1	0.96(0.91, 1.01)	0.82(0.77, 0.87)	0.83(0.79, 0.88)	0.77(0.72, 0.81)	< 0.001	
**Model 2, HR (95% CI)**	1	0.99 (0.94, 1.06)	0.88(0.82, 0.93)	0.90(0.84, 0.96)	0.87(0.81, 0.93)	< 0.001	
**Model 3, HR (95% CI)**	1	1.0(0.95, 1.06)	0.89(0.83, 0.94)	0.91(0.86, 0.97)	0.89(0.83, 0.96)	< 0.001	
**All cancer cause**							
**Mortality rate (per 1000 person-years)**	3.2	3.1	2.5	2.4	2.4		
**Model 1, HR (95% CI)**	1	1.06(0.93, 1.22)	0.83(0.72, 0.96)	0.81(0.70, 0.94)	0.76(0.70, 0.94)	< 0.001	
**Model 2, HR (95% CI)**	1	1.09(0.95, 1.25)	0.87(0.75, 1.01)	0.87(0.75, 1.01)	0.85(0.73, 0.99)	0.002	
**Model 3, HR (95% CI)**	1	1.07(0.93, 1.23)	0.85(0.73, 0.99)	0.83(0.71, 0.98)	0.80(0.67, 0.95)	< 0.001	
**GI cancer cause**							
**Mortality rate (per 1000 person-years)**	1.8	1.9	1.1	1.3	1.3		
**Model 1, HR (95% CI)**	1	1.17(0.97, 1.40)	0.71(0.58, 0.87)	0.81(0.67, 0.99)	0.75(0.61, 0.91)	< 0.001	
**Model 2, HR (95% CI)**	1	1.19(0.99, 1.43)	0.75(0.60, 0.92)	0.87(0.71, 1.07)	0.86(0.70, 1.07)	0.01	
**Model 3, HR (95% CI)**	1	1.17(0.97, 1.41)	0.73(0.59, 0.90)	0.84(0.67, 1.03)	0.82(0.65, 1.03)	0.007	
**CVD cause**							
**Mortality rate (per 1000 person-years)**	7	6.2	5	5.2	5.4		
**Model 1, HR (95% CI)**	1	0.98(0.90, 1.08)	0.82(0.74, 0.90)	0.86(0.78, 0.95)	0.87(0.79, 0.96)	< 0.001	
**Model 2, HR (95% CI)**	1	1.03(0.93, 1.13)	0.87(0.79, 0.97)	0.91(0.82, 1.01)	0.94(0.85, 1.04)	0.04	
**Model 3, HR (95% CI)**	1	1.03(0.94, 1.14)	0.88(0.79, 0.98)	0.91(0.82, 1.02)	0.94(0.83, 1.06)	0.07	
**Other cause**							
**Mortality rate (per 1000 person-years)**	5.9	4.5	4.2	4.6	4.3		
**Model 1, HR (95% CI)**	1	0.86(0.77, 0.95)	0.81(0.73, 0.90)	0.88(0.80, 0.98)	0.77(0.69, 0.86)		< 0.001
**Model 2, HR (95% CI)**	1	0.91(0.82, 1.02)	0.88(0.79, 0.99)	0.98(0.87, 1.09)	0.89(0.79, 1.00)	0.23	
**Model 3, HR (95% CI)**	1	0.92(0.82, 1.02)	0.89(0.80, 1.00)	0.99(0.88, 1.11)	0.91(0.80, 1.04)	0.49	

Model 1: Gender and age adjusted

Model 2: Additionally adjusted for Educational level, Ethnicity, Body mass index, Cigarette smoking, Opiate use, Alcohol consumption, Having a history of diabetes, Having a history of hypertension, Wealth score, Residential area, and Physical activity level

Model 3: Additionally adjusted for Daily energy intake (kcal/d), and Energy-adjusted carbohydrate, protein and fat consumption (g/d)

**Table 4:** Hazard ratios for all-cause and cause-specific mortality, according to quintiles of vegetables intake in a large prospective cohort study with 14 years of follow-up

	**Quintile of Vegetable**					**P for linear trend**
	**1**	**2**	**3**	**4**	**5**	
**Median intake**	98.16	146.49	183.05	224.97	306.51	
**Person-years of follow-up**	129720.060	133681.910	135208.850	135978.590	136974.770	
**All-cause mortality**						
**Mortality rate (per 1000 person-years)**	20.3	16.1	14.6	14.6	14.7	
**Model 1, HR (95% CI)**	1	0.88(0.83, 0.93)	0.84(0.79, 0.89)	0.81(0.77, 0.86)	0.78(0.73, 0.82)	< 0.001
**Model 2, HR (95% CI)**	1	0.95(0.90, 1.00)	0.91(0.86, 0.97)	0.90(0.85, 0.95)	0.90(0.85, 0.96)	< 0.001
**Model 3, HR (95% CI)**	1	0.97(0.92, 1.03)	0.95(0.89, 1.01)	0.94(0.88, 1.00)	0.96(0.90, 1.03)	0.16
**All cancer cause**						
**Mortality rate (per 1000 person-years)**	3.2	2.9	2.6	2.6	2.5	
**Model 1, HR (95% CI)**	1	0.98(0.85, 1.13)	0.92(0.79, 1.06)	0.90(0.78, 1.04)	0.86(0.74, 0.99)	0.02
**Model 2, HR (95% CI)**	1	1.01(0.88, 1.17)	0.96(0.83, 1.10)	0.97(0.84, 1.12)	0.98(0.85, 1.14)	0.60
**Model 3, HR (95% CI)**	1	0.98, (0.85, 1.13)	0.91(0.78, 1.05)	0.90(0.77, 1.05)	0.89(0.75, 1.06)	0.11
**GI cancer cause**						
**Mortality rate (per 1000 person-years)**	1.7	1.6	1.4	1.4	1.2	
**Model 1, HR (95% CI)**	1	1.08(0.90, 1.30)	0.99(0.81, 1.20)	0.95(0.78, 1.16)	0.79(0.65, 0.97)	0.01
**Model 2, HR (95% CI)**	1	1.12(0.93, 1.35)	1.03(0.85, 1.25)	1.03(0.85, 1.26)	0.95(0.77, 1.17)	0.43
**Model 3, HR (95% CI)**	1	1.07(0.88, 1.29)	0.95(0.78, 1.17)	0.94(0.76, 1.16)	0.83(0.66, 1.05)	0.06
**CVD cause**						
**Mortality rate (per 1000 person-years)**	7.8	5.6	4.9	5	5.6	
**Model 1, HR (95% CI)**	1	0.81(0.74, 0.90)	0.76(0.69, 0.84)	0.76(0.69, 0.84)	0.83(0.75, 0.91)	< 0.001
**Model 2, HR (95% CI)**	1	0.90(0.81, 0.99)	0.84(0.76, 0.93)	0.84(0.76, 0.93)	0.94(0.85, 1.03)	0.09
**Model 3, HR (95% CI)**	1	0.93(0.84, 1.02)	0.88(0.80, 0.98)	0.89(0.80, 0.99)	1.01(0.91, 1.13)	0.92
**Other cause**						
**Mortality rate (per 1000 person-years)**	6.1	4.8	4.4	4.2	4.2	
**Model 1, HR (95% CI)**	1	0.88(0.80, 0.98)	0.85(0.76, 0.94)	0.80(0.72, 0.89)	0.77(0.69, 0.86)	< 0.001
**Model 2, HR (95% CI)**	1	0.94(0.84, 1.04)	0.92(0.83, 1.02)	0.88(0.79, 0.98)	0.88(0.79, 0.99)	0.01
**Model 3, HR (95% CI)**	1	0.95(0.85, 1.06)	0.94(0.84, 1.05)	0.90(0.80, 1.01)	0.91(0.80, 1.04)	0.11

Model 1: Gender and age adjusted

Model 2: Additionally adjusted for Educational level, Ethnicity, Body mass index, Cigarette smoking, Opiate use, Alcohol consumption, Having a history of diabetes, Having a history of hypertension, Wealth score, Residential area, and Physical activity level

Model 3: Additionally adjusted for Daily energy intake (kcal/d), and Energy-adjusted carbohydrate, protein and fat consumption (g/d)

The median intake of fiber (13.66, 18.94, 22.49, and 25.67 g/d, and 31.37 g/d, respectively for quintiles 1 to 5), fruits (41.26, 81.74, 120.86, 175.81, and 300.05 g/d, and vegetables (98.16, 146.49, 183.05, 224.97, and 306.51g/d, respectively for quintiles 1 to 5), respectively for quintiles 1 to 5), are also illustrated in Tables 2, 3 and 4.

According to the age and gender-adjusted regression analysis (Model 1), the greater fiber consumption (median intake from about 19 to 31 g/d) was shown to be associated with a lower risk of all-cause mortality by 13- 21% (2^nd^ quintile: HR = 0.87, 95%CI: 0.82, 0.92; 3^rd^ quintile: HR = 0.82, 95%CI: 0.77, 0.87; 4^th^ quintile: HR = 0.83, 95%CI: 0.78, 0.88; and 5^th^ quintile: HR = 0.79, 95%CI: 0.74, 0.84), CVD mortality by 11–14% (2^nd^ quintile: HR = 0.89, 95%CI: 0.80, 0.97; 3^rd^ quintile: HR = 0.86, 95%CI: 0.78, 0.95; 4^th^ quintile: HR = 0.89, 95%CI: 0.81, 0.99; and 5^th^ quintile: HR = 0.88, 95%CI: 0.80, 0.98), and other cause mortality 21–23% (2^nd^ quintile: HR = 0.79, 95%CI: 0.71, 0.87; 3^rd^ quintile: HR = 0.79, 95%CI: 0.71, 0.88; 4^th^ quintile: HR = 0.79, 95%CI: 0.70, 0.88; and 5^th^ quintile: HR = 0.77, 95%CI: 0.69, 0.86), compared to the lowest fiber intake as the reference category (median intake ~ 14 g/d) (Table 2).

When taking into account the effects of additional confounding variables including educational level, ethnicity, BMI, cigarette smoking, opiate use, alcohol consumption, history of diabetes, history of hypertension, wealth score, residential area, and physical activity level in Model 2 of Cox regression analysis, similar findings were noted as those with greater dietary fiber intake were found to have a lower risk of all-cause by nearly 9–13% (2^nd^ quintile: HR = 0.91, 95%CI: 0.86, 0.96; 3^rd^ quintile: HR = 0.88, 95%CI: 0.83, 0.93; 4^th^ quintile: HR = 0.91, 95%CI: 0.86, 0.97; and 5^th^ quintile: HR = 0.87, 95%CI: 0.82, 0.92), and a reduced risk for other cause of death (2^nd^ quintile: HR = 0.84, 95%CI: 0.75, 0.93; 3^rd^ quintile: HR = 0.87, 95%CI: 0.78, 0.97; 4^th^ quintile: HR = 0.88, 95%CI: 0.79, 0.99; and 5^th^ quintile: HR = 0.86, 95%CI: 0.77, 0.96), when comparing with the first quintile of fiber intake (Table 2).

**Fig. 1:**
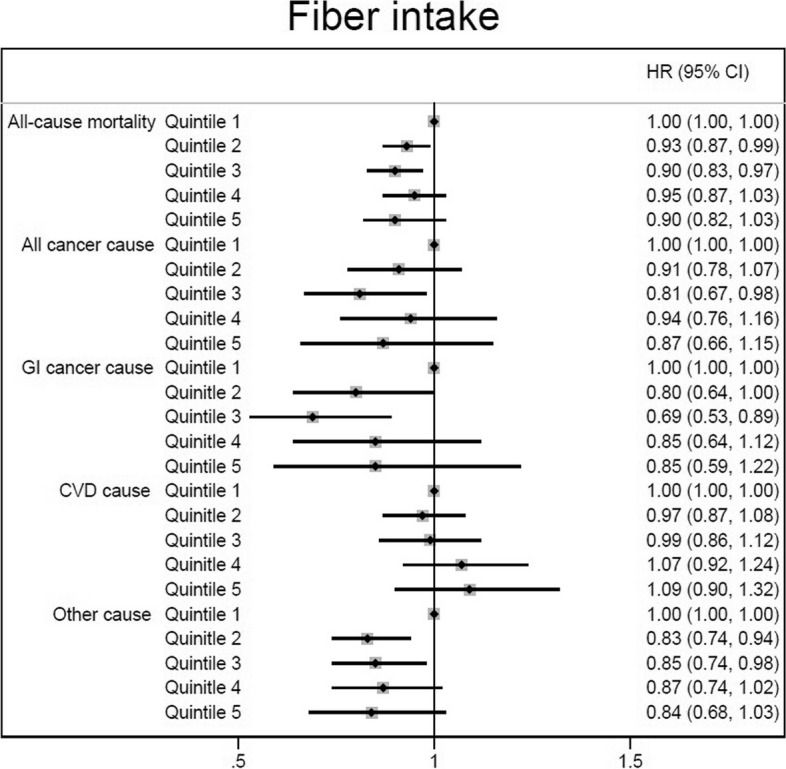
The association between dietary fiber intake (quintiles 2–5 versus 1) and all-cause and cause-specific mortality according to fully adjusted Cox regression analysis (Model 3 adjusted for gender, age, educational level, ethnicity, body mass index, cigarette smoking, opiate use, alcohol consumption, having a history of diabetes, having a history of hypertension, wealth score, residential area, physical activity level, and dietary data including daily energy intake (kcal/d), and energy-adjusted carbohydrate, protein and fat consumption)

**Fig. 2:**
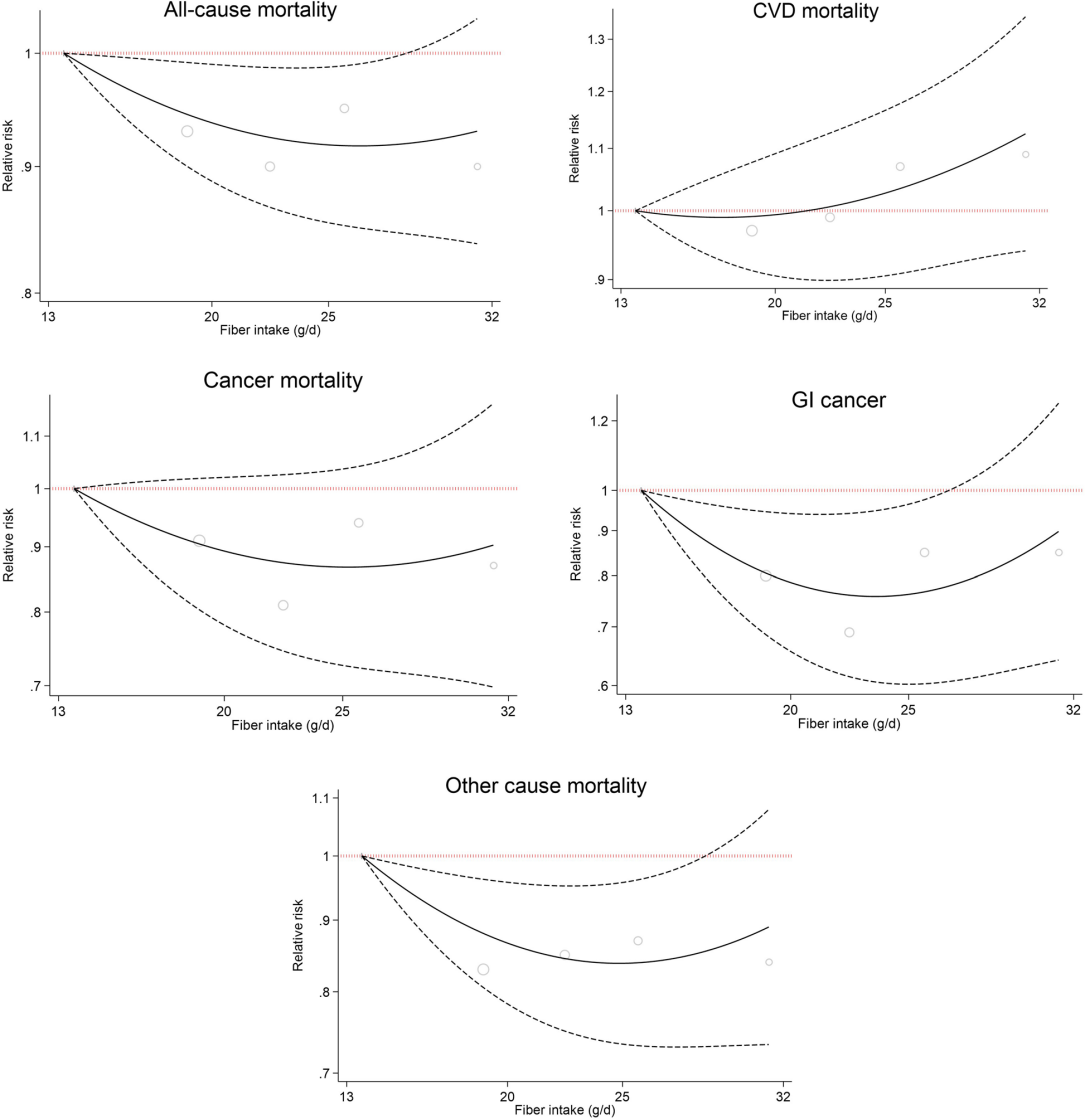
Association between different amounts of fiber intake and death from all-cause, CVD, all cancer, GI cancer, and other cause. CVD, cardiovascular disease; GI, gastrointestinal

However, after additionally controlling for daily energy, and macronutrients intakes in the Model 3 of Cox regression analysis, only those in the second and third quintiles of dietary fiber intake showed a reduced risk of all-cause (by 7–10%) and other cause of death (by 15%-17%) (for all-cause mortality: 2^nd^ quintile: HR = 0.93, 95%CI: 0.87, 0.99; and 3^rd^ quintile: HR = 0.90, 95%CI: 0.83, 0.97; for other cause of death: 2^nd^ quintile: HR = 0.83, 95%CI: 0.74, 0.94; and 3^rd^ quintile: HR = 0.85, 95%CI: 0.74, 0.98), while the overall associations were not statistically significant (Table 2 and Fig. 1). The dose–response relationship was non-significant for all of the outcomes (Fig. 2).

### Fruits intake and all-cause and cause-specific mortality

When total fruits consumption was explored, it was observed that according to Model 1, increasing intakes of fruits from the third to fifth quintiles (median intake from 121 to 300 g/d) was linked to a lower risk of all-cause mortality (3^rd^ quintile: HR = 0.82, 95%CI: 0.77, 0.87; 4^th^ quintile: HR = 0.83, 95%CI: 0.79, 0.88; and 5^th^ quintile: HR = 0.77, 95%CI: 0.72, 0.81), all cancer mortality (3^rd^ quintile: HR = 0.83, 95%CI: 0.72, 0.96; 4^th^ quintile: HR = 0.81, 95%CI: 0.70, 0.94; and 5^th^ quintile: HR = 0.76, 95%CI: 0.70, 0.94), GI cancer mortality (3^rd^ quintile: HR = 0.71, 95%CI: 0.58, 0.87; 4^th^ quintile: HR = 0.81, 95%CI: 0.67, 0.99; and 5^th^ quintile HR = 0.75, 95%CI: 0.61, 0.91), CVD mortality (3^rd^ quintile: HR = 0.82, 95%CI: 0.74, 0.90; 4^th^ quintile: HR = 0.86, 95%CI: 0.78, 0.95; and 5^th^ quintile HR = 0.87, 95%CI: 0.79, 0.96), and other cause mortality (3^rd^ quintile: HR = 0.81, 95%CI: 0.73, 0.90; 4^th^ quintile: HR = 0.88, 95%CI: 0.80, 0.98; and 5^th^ quintile HR = 0.77, 95%CI: 0.69, 0.86) compared to those in the lowest quintile consuming about 41 g/d of fruits (Table 3).

In Model 2 of Cox regression analysis, additional adjustment for other confounding variables revealed that increasing the intake of fruits was accompanied by 10–13% reduced risk for all-cause mortality in comparison with the lowest intake (3^rd^ quintile: HR = 0.88, 95%CI: 0.82, 0.93; 4^th^ quintile: HR = 0.90, 95%CI: 0.84, 0.96; and 5^th^ quintile HR = 0.87, 95%CI: 0.81, 0.93). Besides, the subjects in the last quintile of fruits demonstrated a lower risk of all cancer mortality by 15% (HR = 0.85, 95%CI: 0.73, 0.99) compared to those in the first quintile (Table 3).

**Fig. 3:**
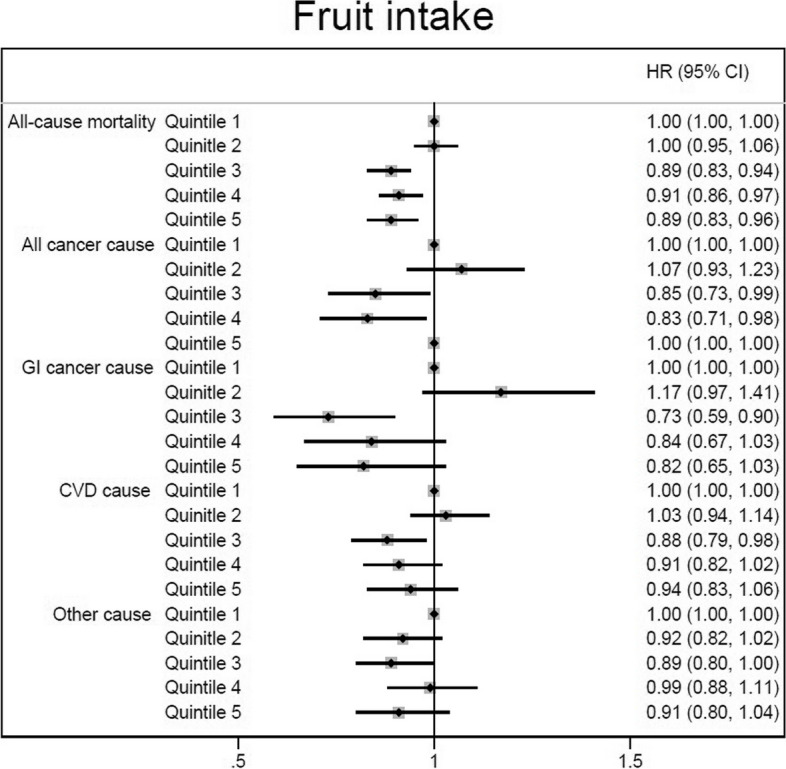
The association between dietary fruits intake (quintiles 2–5 versus 1) and all-cause and cause-specific mortality according to fully adjusted Cox regression analysis (Model 3 adjusted for gender, age, educational level, ethnicity, body mass index, cigarette smoking, opiate use, alcohol consumption, having a history of diabetes, having a history of hypertension, wealth score, residential area, physical activity level, and dietary data including daily energy intake (kcal/d), and energy-adjusted carbohydrate, protein and fat consumption)

**Fig. 4:**
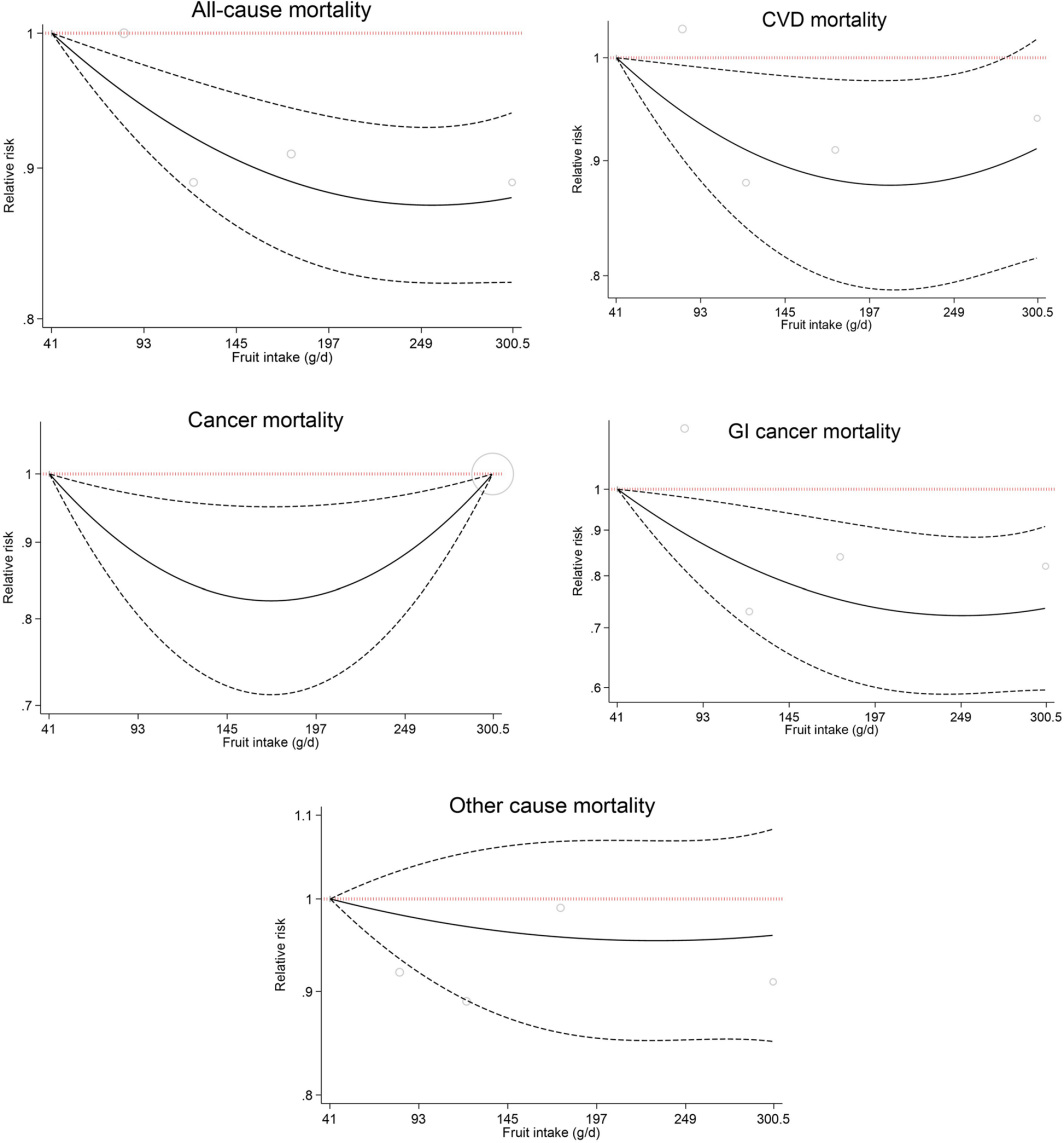
Association between different amounts of fruit intake and death from all-cause, CVD, all cancer, GI cancer, and other cause. CVD, cardiovascular disease; GI, gastrointestinal

Following taking into consideration the daily energy and macronutrients intakes in addition to other potentials variables in Cox regression Model 3, increasing consumption of fruits was associated with a decreased risk of mortality for all-cause mortality by 9–11% (3^rd^ quintile: HR = 0.89, 95%CI: 0.83, 0.94; 4^th^ quintile: HR = 0.91, 95%CI: 0.86, 0.97; and 5^th^ quintile HR = 0.89, 95%CI: 0.83, 0.96), and all cancer cause mortality by 15–20% (3^rd^ quintile: HR = 0.85, 95%CI: 0.73, 0.99; 4^th^ quintile: HR = 0.83, 95%CI: 0.71, 0.98; and 5^th^ quintile HR = 0.80, 95%CI: 0.67, 0.95) compared to the lowest intake. Also, a significant decreasing trend was observed across quintiles of fruits consumption for GI cancer mortality risk (Table 3 and Fig. 3).A significant dose response relationship was observed for death from all-cause, all cancer and GI cancer, and a marginally significant relationship for CVD mortality. The relationship between fruit intake and other cause of death was not dose–response (Fig. 4).

### Vegetables intake and all-cause and cause-specific mortality

Increasing vegetables consumption from about 146 to 307 g/d as compared to 98 g/d was found to reduce the risk of mortality from all-cause (2^nd^ quintile: HR = 0.88, 95%CI: 0.83, 0.93; 3^rd^ quintile: HR = 0.84, 95%CI: 0.79, 0.89; 4^th^ quintile: HR = 0. 81, 95%CI: 0.77, 0.86; and 5^th^ quintile: HR = 0.78, 95%CI: 0.73, 0.82), CVD (2^nd^ quintile: HR = 0.81, 95%CI: 0.74, 0.90; 3^rd^ quintile: HR = 0.76, 95%CI: 0.69, 0.84; 4^th^ quintile: HR = 0.76, 95%CI: 0.69, 0.84; and 5^th^ quintile: HR = 0.83, 95%CI: 0.75, 0.91), and other cause (2^nd^ quintile: HR = 0.88, 95%CI: 0.80, 0.98; 3^rd^ quintile: HR = 0.85, 95%CI: 0.76, 0.94; 4^th^ quintile: HR = 0.80, 95%CI: 0.72, 0.89; and 5^th^ quintile: HR = 0.77, 95%CI: 0.69, 0.86, compared to the first quintile as the refrence category) (Table 4).

This inverse relationship remained significant after controlling for additional confounding variables in Model 2 of Cox regression analysis for all-cause and other cause mortality. The risk of all-cause mortality was reduced by almost 9–10% by increasing the intake of vegetables (3^rd^ quintile: HR = 0.91, 95%CI: 0.86, 0.97; 4^th^ quintile: HR = 0.90, 95%CI: 0.85, 0.95; and 5^th^ quintile: HR = 0.90, 95%CI: 0.85, 0.96) in comparison with the lowest vegetables consumption. Furthermore, greater vegetables consumption showed a decreased risk of CVD mortality by 10–16%, and (3^rd^ quintile: HR = 0.90, 95%CI: 0.81, 0.99; 4^th^ quintile: HR = 0.84, 95%CI: 0.76, 0.93 and 5^th^ quintile: HR = 0.84, 95%CI: 0.76, 0.93) for those in the fifth quintiles of vegetables intake compared to the first quintile. The risk of other cause of death was also demonstrated to reduce by 12% for those in the third and fourth quintiles of vegetables consumption in comparison with the lowest quintile (3^rd^ quintile: HR = 0.88, 95%CI: 0.79, 0.98 and 4^th^ quintile: HR = 0.88, 95%CI: 0.79, 0.99) (Table 4).

**Fig. 5:**
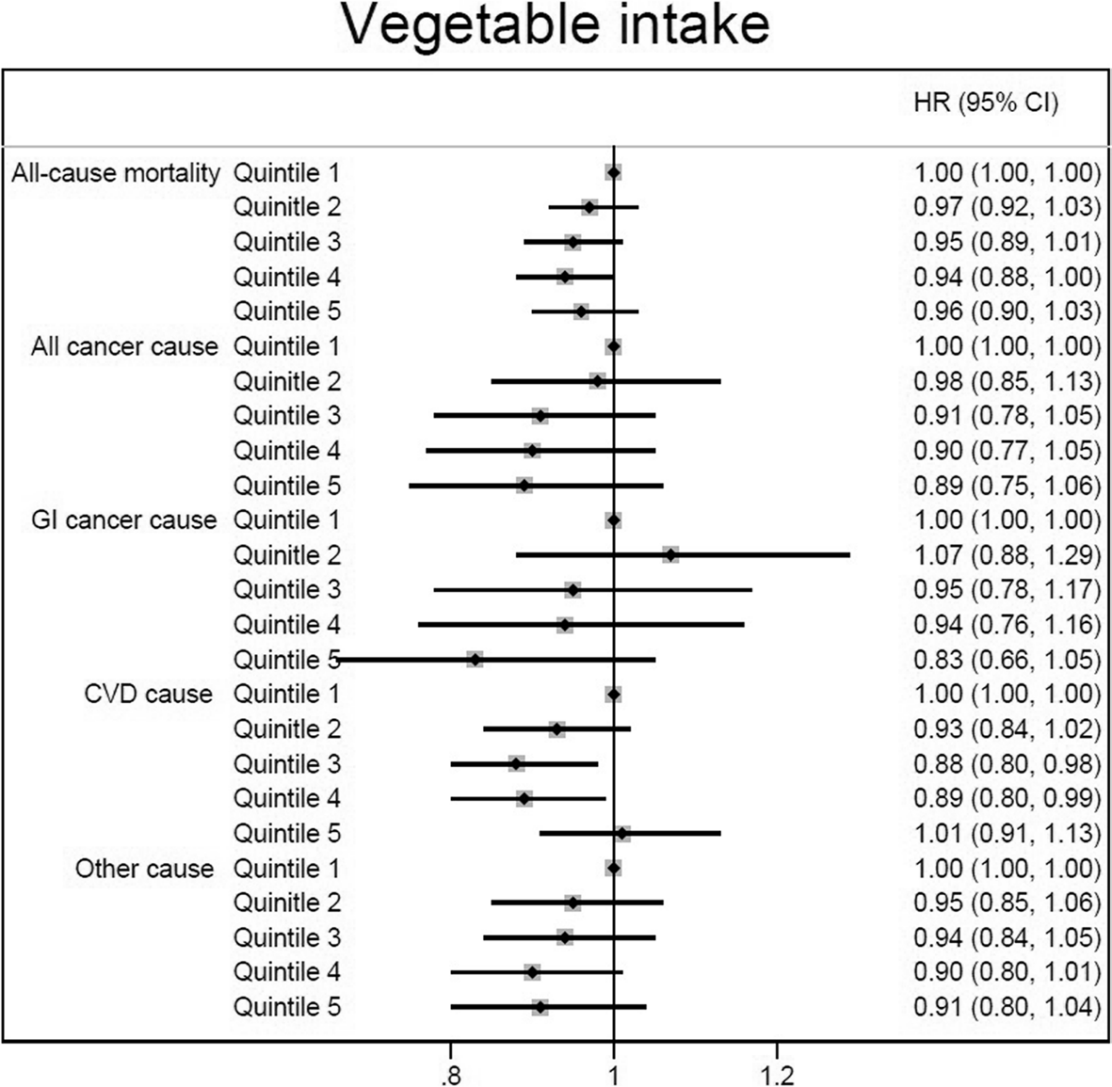
The association between dietary vegetables intake (quintiles 2–5 versus 1) and all-cause and cause-specific mortality according to fully adjusted Cox regression analysis (Model 3 adjusted for gender, age, educational level, ethnicity, body mass index, cigarette smoking, opiate use, alcohol consumption, having a history of diabetes, having a history of hypertension, wealth score, residential area, physical activity level, and dietary data including daily energy intake (kcal/d), and energy-adjusted carbohydrate, protein and fat consumption)

**Fig. 6:**
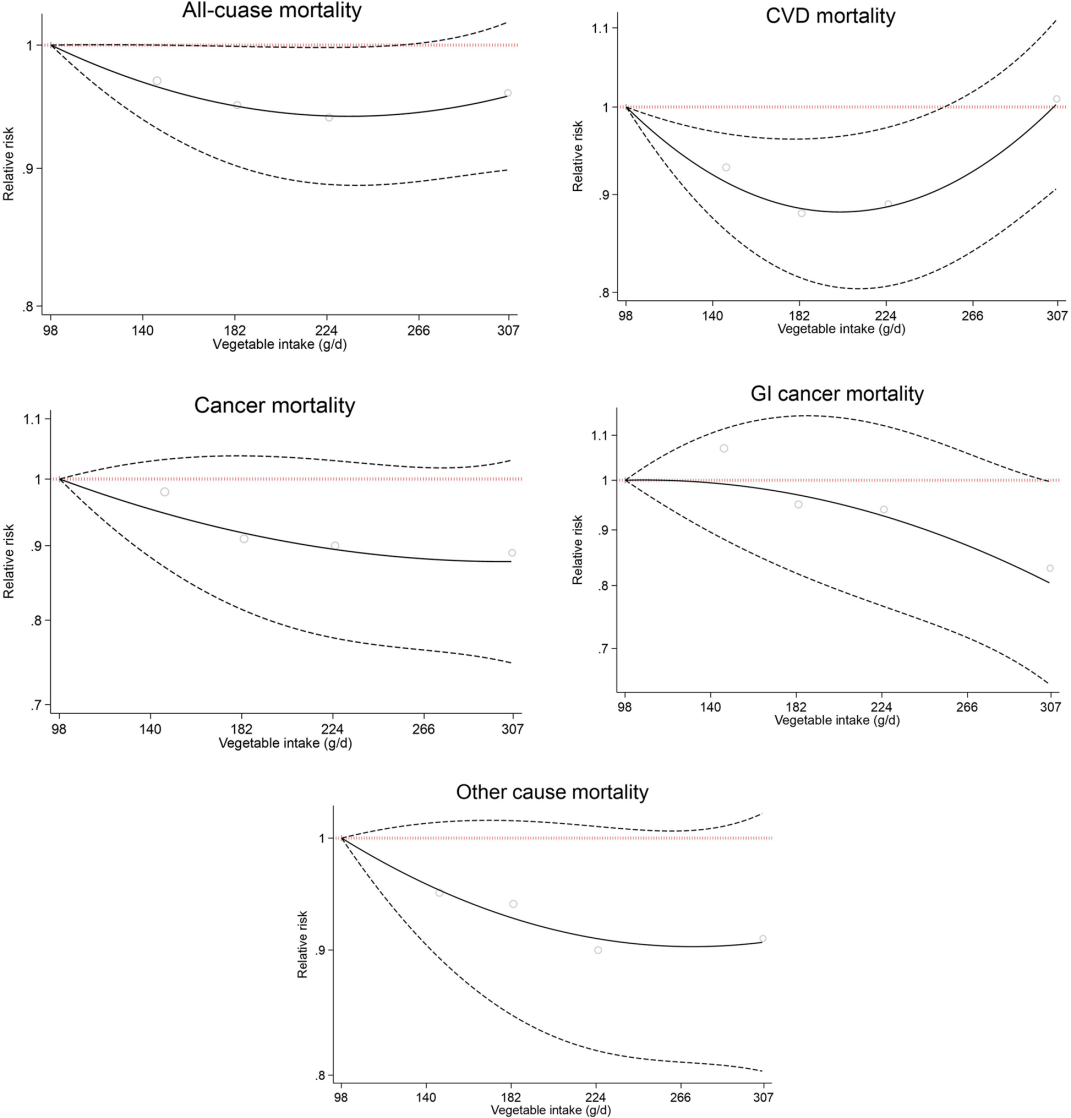
Association between different amounts of vegetable intake and death from all-cause, CVD, all cancer, GI cancer, and other cause. CVD, cardiovascular disease; GI, gastrointestinal

However, after considering the daily energy and macronutrients intake in the fully adjusted Cox regression model (Model 3), it failed to detect these associations as significant, except that those in the third and fourth quintiles of vegetables intake were found to have 11–12% lower risk of CVD mortality (3^rd^ quintile: HR = 0.88, 95%CI: 0.80, 0.98; 4^th^ quintile: HR = 0.89, 95%CI: 0.80, 0.99; compared to the first quintile as the reference category) (Table 4 and Fig. 5). No significant dose–response relationship was observed between vegetable intake and death from all-cause, CVD, all cancer, and other cause. Only a marginally significant dose–response relationship was observed for GI cancer (Fig. 6).

### Sensitivity analysis

The sensitivity analysis indicated that according to the multivariable Cox regression Model 3, the inverse association between higher intake of dietary fiber (2^nd^ and 3^rd^ quintiles versus 1^st^ quintile) and lower all-cause mortality risk became statistically non-significant following excluding the patients with a history of chronic diseases,: 2nd quintile: HR = 0.93, 95%CI: 0.84, 1.02; and 3rd quintile: HR = 0.90, 95%CI: 0.80, 1.01 (Supplementary Table 1). Similar results were observed when Smokers, opium users and alcohol drinkers were excluded; such that the significant relationship in the 2nd and 3rd quintiles became non-significant (2nd quintile: HR = 1.00, 95%CI: 0.92, 1.09; and 3rd quintile: HR = 0.98, 95%CI: 0.89, 1.09) (Supplementary Table 1).

The multivariable-adjusted HRs for all-cause mortality according to quintiles of fruits and vegetables consumption remained consistent after we excluded the patients with chronic diseases, extreme BMI, those with a history of smoking, opium or alcohol use, or the subjects who were followed-up in the first 2 years of study (Supplementary Tables 2 and 3).

## Discussion

The current findings from the GCS, a large prospective cohort study on 48,632 subjects who were followed for about 14 years on average, demonstrated an inverse relationship between increasing consumption of dietary fiber and fruits and reducing all-cause mortality risk after controlling for the effects of potential confounders and dietary intakes. Regarding cause-specific death risk, it was observed that increasing the intake of vegetables was accompanied by a reduced risk of CVD mortality. Also, higher fruit consumption was linked to a lower all-cancers mortality risk. Besides, greater fiber intake was associated with a decreased risk of mortality due to other causes. These findings further support the current national recommendations on following a healthy diet containing proper amounts of these health-protective dietary items. However, as revealed by the sensitivity analysis, the inverse association between higher intake of dietary fiber (2nd and 3rd quintiles versus 1st quintile) and lower all-cause mortality risk can only be generalized to healthy individuals who do not have a history of chronic diseases or smoking, opium or alcohol use.

Several studies have indicated a link between the consumption of fiber, fruits, and vegetables and a reduced risk of mortality [16, 19–23, 38–40]. For instance, an Australian cohort study found an inverse association between fruit and vegetable intake and all-cause mortality [16]. This was also observed in relation to fresh fruits, root vegetables, and fruiting vegetables in a Spanish cohort [38]. Studies have also reported protective effects of fruits and vegetables consumption regarding cause-specific mortality [19, 21, 40–45]. A study in Norway found that higher intakes of vegetables, fruits, and berries were inversely associated with all-cause, cancer-cause, and stroke-cause mortality risk [41]. The HAPIEE study found that fruit and vegetable consumption was associated with reduced CVD mortality among smokers and patients with hypertension [42]. Higher vegetables consumption, particularly cruciferous vegetables and fruits intakes, were inversely associated with all-cause and CVD-cause mortality risk in a dose–response manner [43]. According to another population-based cohort study, fruits and vegetables intake of fewer than five servings per day is associated with progressively shorter survival and higher mortality rates [44]. In the previously mentioned prospective cohort study, vegetables consumption of 500 gr/day and more was associated with a 33% lower risk of cancer-cause mortality compared to lower than 100 gr/day consumption [40]. The nationwide China Kadoorie Biobank Study has reported a dose–response association between fruits intake for four days per week and more with lower all-cause, cancer-cause, and CVD-cause mortality [45]. In the current large prospective cohort study, increasing consumption of fruits from a median of 121 to 300 g/d was associated with a decreased risk of mortality due to all-cause and all cancer by about 9–11%, and 15–20%, respectively when compared to those who consume a median of 41 g/d of fruits. However, no significant associations were found for greater vegetable consumption and reducing all-cause and cause-specific death risk after considering daily energy and macronutrients consumption; except those who consume approximately 183 to 225 g/d of vegetables were found to have 11–12% lower risk of death due to CVD causes than those with an intake of about 98 g/d.

There is strong evidence that increasing the consumption of vegetables and fruits can reduce the risk of hypertension, coronary heart disease, and stroke. It is also likely that the risk of cancer is inversely related to the consumption of vegetables and fruits. Furthermore, there is some evidence to suggest that increasing the consumption of vegetables and fruits may help prevent weight gain [46]. Fruits and vegetables intake provide health benefits through several ways including the antioxidant capacity of vitamins and minerals, the effect of folate and other B vitamins on homocysteine levels, and presence of detoxifying enzymes [47, 48]. Besides, fruits and vegetables contain large amounts of polyphenols with anti-inflammatory, vasodilator and anti-thrombosis properties [49]. Based on the epidemiological surveys fruits and vegetables intake may be associated with risk of specific cancers than all-type cancers and its preventive role is dominantly through the digestive system [7]. On the other hand, the antioxidant capacity of vegetables and fruits may affect arterial stiffness [50]. The anti-hypertensive role of fruits and vegetables due to a high content of potassium and magnesium may demonstrate its cardio-protective effects [51]. Fiber is another component in fruits and vegetables that could be the responsible for demonstrated results.

As shown in the current study, an overall inverse trend was found across the quintiles of fiber when it is investigated in relation to all-cause, all cancer and GI cancer death risk, though it was not statistically significant. Specifically, the results revealed that the studied participants who consume about 19–22 g of fiber per day had a significantly reduced risk of death due to all-cause (by about 7–10%) and other cause (by about 15–17%) when comparing to those whose daily fiber intake was about 14 g. Similarly, according to a large European prospective study among 452,717 participants, it was revealed that fiber intake is associated with a reduced hazard for total mortality [19]. Besides, the results of the Japan Public Health Center-based study showed that fiber intake was associated with a reduced all-cause mortality [20]. In a large US cohort study intakes of total fiber, soluble fiber, and insoluble fiber were inversely associated with hazards of all-cause mortality [21]. The PREDIMED study has revealed inverse association between intakes of fiber and fruit and total mortality [22]. The SUN project has also reported an inverse association between total dietary fiber, particularly vegetables fiber, with all-cause mortality in the Mediterranean population [23]. In the aforementioned European prospective study, fiber intake was associated with a reduced circulatory, digestive, and non-CVD non-cancer inflammatory diseases mortality [19]. In the previously mentioned US cohort study, intakes of total fiber, soluble fiber, and insoluble fiber were also inversely associated with hazards of cardiovascular and cancer cause mortality [21].

Fiber alone or as a component of fruits and vegetables has several benefits which show its role in reducing all-cause and cause-specific mortality. Fiber intake may help to lower serum cholesterol concentrations by increasing the fecal excretion of bile acids in feces [52]. Fiber fermentation by gut microbiota results in short-chain fatty acids (SCFAs) production which inhibit hepatic fatty acid synthesis [53]. Besides, SCFAs reduce gut permeability to endotoxin which prevents systemic inflammation [54]. Fiber consumption results in slower digestion and more sustained satiety which leads to body weight control [55]. Besides, it may reduce systolic and diastolic blood pressure [56], be associated with a reduced risk of type 2 diabetes mellitus by increasing insulin sensitivity [57] and is shown to be inversely associated with inflammatory marcers [58] including C-reactive protein, interleukin-6, and tumor necrosis factor-α [58–60] which all are health-affecting factors.

However, inconsistencies found in some studies compared to the current results regarding fruit consumption. In a cohort study among European Population with diabetes, vegetables intake was associated with a significantly reduced all-cause mortality risk; however, this inverse association was non-significant for fruits consumption [39]. Another prospective cohort study showed an indirect association between vegetables consumption and cancer-cause mortality that was not seen for fruits intake [40]. Fresh fruits, in addition to sucrose and high fructose corn syrup, contain fructose [61]. Excessive sugar consumption is increasingly being viewed as a contributor to the growing epidemics of obesity and related cardiometabolic diseases. Fructose metabolism and the properties of fructose-derived metabolites allow fructose to act as a physiological signal of normal dietary sugar consumption. However, when consumed in excess, these unique properties may contribute to the development of cardiometabolic disease. Fructose is quickly absorbed by the intestines and liver, where it is used for energy, converted into glucose and its storage form glycogen, or turned into fatty acids and stored as triglycerides [62]. Fructose consumption has been increasingly scrutinized as a potential cause of hyperuricemia, as urate is produced as a byproduct of fructose metabolism [61]. Therefore, it is important to pay attention to the amount of fruit consumed per day, taking into account the individual’s calorie intake.

Long time follow-up in the current study was one of the strengths which allowed us to explore cause-specific mortality other than all-cause mortality. Besides, enrolling a large sample size can be considered as another strength. An extensive range of demographic data collection other than age and gender led to run adjusted model based on possible confounders. A valid and reliable FFQ was used to collect dietary data. Although residual confounding risk by a general healthy lifestyle of is yet probable as shown before [63].

## Conclusion

The current findings from the GCS, a large prospective cohort study on about 48632 subjects who were followed by about 14 years on average, demonstrated protective effects of increasing the consumption of dietary fiber, and fruits against all-cause mortality risk following controlling for the effects of potential confounders and dietary intakes. It was also observed that increasing the intake of vegetables was accompanied by a reduced risk of CVD mortality. Higher fruits consumption was linked to a lower all-cancers mortality risk. Besides, greater fiber intake contributed to with a decreased risk of mortality due to other causes. These findings would further support the current national recommendations on following a healthy diet containing proper amounts of these health-protective dietary items. However, additional large sample size cohort studies with long term follow-up duration are required to establish these findings particularly regarding the cause-specific mortality risk. Also, well-designed experimental studies would be helpful in clarifying the predominant mechanisms responsible for the protective effects of fruits, vegetables, and fiber against mortality.

### Supplementary Information

**Additional file 1.** Supplementary materials.

## Data Availability

The datasets analyzed during the current study are available from the corresponding author on reasonable request.

## Abbreviations

CVD: Cardiovascular disease
GBD: Global Burden of Disease
WHO: World health organization
EPIC: The European Prospective Investigation into Cancer and Nutrition
PREDIMED: The Prevención con Dieta Mediterránea study
SUN: The "Seguimiento Universidad de Navarra" project
GCS: Golestan Cohort Study
EC: Esophageal cancer
FFQ: Food frequency questionnaire
SES: Socio-economic status
SDs: Standard deviations
IQR: Interquartile range
WS: Wealth score
HRs: Hazard ratios
HAPIEE: The Health, Alcohol and Psychosocial Factors in Eastern Europe study
SCFAs: Short-chain fatty acids
