# Supplementary material for: Higher intakes of fiber, total vegetables, and fruits may attenuate the risk of all-cause and cause-specific mortality: findings from a large prospective cohort study
Source: Nutr J. 2023 Nov 17;22:60. doi: 10.1186/s12937-023-00883-4 (PMC10655472; doi:10.1186/s12937-023-00883-4)
Supplement: Supplementary file 1 — Additional file 1. Supplementary materials. [file 12937_2023_883_MOESM1_ESM.docx]

**Supplementary materials:**

**Supplementary Table 1. Sensitivity analyses of the association between dietary fiber intake and all-cause mortality in a large prospective cohort study with 14 years of follow-up.**

|  | **Quintile of Fiber** | | | | | **P for linear trend** |
| --- | --- | --- | --- | --- | --- | --- |
|  | **1** | **2** | **3** | **4** | **5** |  |
| **Patients with chronic diseases were excluded** | | | | | |  |
| Person-years of follow-up | 87909.3 | 94608.4 | 100859.3 | 103352.8 | 105588.2 |  |
| Mortality rate (per 1000 person-years) | 13.37 | 10.79 | 10.08 | 9.83 | 10.67 |  |
| Multivariable-adjusted hazard ratio (95% CI) | 1.00 | 0.93 (0.84, 1.02) | 0.90 (0.80, 1.01) | 0.96 (0.84, 1.09) | 0.96 (0.81, 1.14) | 0.794 |
| **Participants with extreme BMI were excluded** | | | | | | |
| Person-years of follow-up | 112083.1 | 117917.1 | 118939.8 | 121327.4 | 124638.1 |  |
| Mortality rate (per 1000 person-years) | 19.24 | 15.93 | 14.33 | 14.29 | 14.47 |  |
| Multivariable-adjusted hazard ratio (95% CI) | 1.00 | 0.93 (0.87, 0.99) | 0.91 (0.84, 0.99) | 0.94 (0.86, 1.03) | 0.90 (0.79, 1.01) | 0.159 |
| **Smokers, opium users and alcohol drinkers were excluded** | | | | | | |
| Person-years of follow-up | 102095.5 | 104809.7 | 103781.2 | 102806.3 | 95700.2 |  |
| Mortality rate (per 1000 person-years) | 10.26 | 8.8 | 8.83 | 9.02 | 10.59 |  |
| Multivariable-adjusted hazard ratio (95% CI) | 1.00 | 1.0 (0.92, 1.09) | 0.98 (0.89, 1.09) | 1.03 (0.92, 1.16) | 1.03 (0.89, 1.20) | 0.598 |
| **First 2 years of follow-up were excluded** | | | | | | |
| Person-years of follow-up | 129728.5 | 133316.2 | 134656.6 | 136001.1 | 137861.7 |  |
| Mortality rate (per 1000 person-years) | 19.86 | 16.39 | 14.64 | 14.51 | 15 |  |
| Multivariable-adjusted hazard ratio (95% CI) | 1.00 | 0.93 (0.87, 0.99) | 0.90 (0.83, 0.97) | 0.94 (0.86, 1.03) | 0.92 (0.81, 1.03) | 0.251 |
| Multivariable fully adjusted Cox regression analysis  Chronic disease including a previous cancer, diabetes or hypertension.  Extreme BMIs were considered as < 18.5 or > 35 kg/m2 | | | | | | |

**Supplementary Table 2. Sensitivity analyses of the association between fruit intake and all-cause mortality in a large prospective cohort study with 14 years of follow-up.**

|  | **Quintile of Fruit** | | | | | **P for linear trend** |
| --- | --- | --- | --- | --- | --- | --- |
|  | **1** | **2** | **3** | **4** | **5** |  |
| **Patients with chronic diseases were excluded** | | | | | |  |
| Person-years of follow-up | 95070.17 | 95711.62 | 98052.61 | 99540.72 | 103943 |  |
| Mortality rate (per 1000 person-years) | 13.73 | 11.34 | 9.83 | 9.92 | 9.76 |  |
| Multivariable-adjusted hazard ratio (95% CI) | 1.00 | 0.98 (0.90, 1.07) | 0.88 (0.80, 0.96) | 0.86 (0.78, 0.95) | 0.82 (0.74, 0.91) | <0.001 |
| **Participants with extreme BMI were excluded** | | | | | | |
| Person-years of follow-up | 112978.9 | 115831.1 | 119610 | 120875.7 | 125609.7 |  |
| Mortality rate (per 1000 person-years) | 18.75 | 16.53 | 14.02 | 14.59 | 14.35 |  |
| Multivariable-adjusted hazard ratio (95% CI) | 1.00 | 1.01 (0.95, 1.08) | 0.89 (0.84, 0.96) | 0.93 (0.87, 0.99) | 0.91 (0.84, 0.98) | 0.001 |
| **Smokers, opium users and alcohol drinkers were excluded** | | | | | | |
| Person-years of follow-up | 100431.6 | 102151.8 | 104147.4 | 102417.9 | 100044.2 |  |
| Mortality rate (per 1000 person-years) | 12.14 | 9.71 | 8.29 | 8.42 | 8.89 |  |
| Multivariable-adjusted hazard ratio (95% CI) | 1.00 | 1.01 (0.94, 1.09) | 0.88 (0.82, 0.95) | 0.91 (0.84, 0.99) | 0.91 (0.83, 0.99) | 0.004 |
| **First 2 years of follow-up were excluded** | | | | | | |
| Person-years of follow-up | 129310.9 | 131690.2 | 134424.1 | 135841 | 140298 |  |
| Mortality rate (per 1000 person-years) | 19.58 | 17 | 14.4 | 14.89 | 14.58 |  |
| Multivariable-adjusted hazard ratio (95% CI) | 1.00 | 1.00 (0.95, 1.06) | 0.89 (0.83, 0.94) | 0.91 (0.6, 0.97) | 0.89 (0.83, 0.96) | 0.001 |
| Multivariable fully adjusted Cox regression analysis  Chronic disease including a previous cancer, diabetes or hypertension.  Extreme BMIs were considered as < 18.5 or > 35 kg/m2 | | | | | | |

**Supplementary Table 3. Sensitivity analyses of the association between vegetable intake and all-cause mortality in a large prospective cohort study with 14 years of follow-up.**

|  | **Quintile of Vegetable** | | | | | **P for linear trend** |
| --- | --- | --- | --- | --- | --- | --- |
|  | **1** | **2** | **3** | **4** | **5** |  |
| **Patients with chronic diseases were excluded** | | | | | |  |
| Person-years of follow-up | 91156.29 | 99306.22 | 101180.6 | 100630.6 | 100044.5 |  |
| Mortality rate (per 1000 person-years) | 13.45 | 11.24 | 9.93 | 10.04 | 9.99 |  |
| Multivariable-adjusted hazard ratio (95% CI) | 1.00 | 0.98 (0.90, 1.07) | 0.94 (0.86, 1.03) | 0.94 (0.86, 1.04) | 0.97 (0.87, 1.07) | 0.399 |
| **Participants with extreme BMI were excluded** | | | | | | |
| Person-years of follow-up | 114056.3 | 118752.2 | 119891.6 | 121047.1 | 121158.3 |  |
| Mortality rate (per 1000 person-years) | 19.67 | 15.63 | 14.13 | 14.13 | 14.63 |  |
| Multivariable-adjusted hazard ratio (95% CI) | 1.00 | 0.97(0.91, 1.04) | 0.95(0.89, 1.01) | 0.93(0.87, 1.0) | 0.98(0.91, 1.05) | 0.319 |
| **Smokers, opium users and alcohol drinkers were excluded** | | | | | | |
| Person-years of follow-up | 97665.35 | 101231.8 | 104375.2 | 102963 | 102957.6 |  |
| Mortality rate (per 1000 person-years) | 11.4 | 9.99 | 8.67 | 8.87 | 8.58 |  |
| Multivariable-adjusted hazard ratio (95% CI) | 1.00 | 0.98(0.91, 1.05) | 0.91 (0.84, 0.99) | 0.93 (0.86, 1.01) | 0.92 (0.4, 1.00) | 0.035 |
| **First 2 years of follow-up were excluded** | | | | | | |
| Person-years of follow-up | 129720.1 | 133681.9 | 135208.8 | 135978.6 | 136974.8 |  |
| Mortality rate (per 1000 person-years) | 20.34 | 16.12 | 14.64 | 14.65 | 14.67 |  |
| Multivariable-adjusted hazard ratio (95% CI) | 1.00 | 0.97 (0.92, 1.03) | 0.95 (0.89, 1.00) | 0.94 (0.88, 1.00) | 0.96 (0.90, 1.03) | 0.128 |
| Multivariable fully adjusted Cox regression analysis  Chronic disease including a previous cancer, diabetes or hypertension.  Extreme BMIs were considered as < 18.5 or > 35 kg/m2 | | | | | | |
